# Supplementary material for: The carbon perception gap in actual and ideal carbon footprints across wealth groups
Source: Nat Commun. 2025 Jul 4;16:6180. doi: 10.1038/s41467-025-61505-7 (PMC12227583; doi:10.1038/s41467-025-61505-7)
Supplement: Supplementary file 1 — Supplementary Information [file 41467_2025_61505_MOESM1_ESM.pdf]

Supplementary Information:

The carbon perception gap in actual and ideal carbon footprints across wealth groups

Johanna Köchling[*,+,1,2], Julia E. Koller[+,1], Jana Straßheim[1,2], Yannic Rehm[3], Lucas Chancel[4,3], Claudia Diehl[5], Harald T. Schupp[1,2], Britta Renner[1,2]

[1] Department of Psychology, University of Konstanz, Universitätsstraße 10, 78464 Konstanz, Germany, [2] Centre for the Advanced Study of Collective Behaviour, University of Konstanz, Universitätsstraße 10, 78464 Konstanz, Germany, [3] Paris School of Economics, 48 boulevard Jourdan, 75014 Paris, France, [4] Center for Research on Social Inequalities, Sciences Po, 27 rue Saint Guillaume, 75337 Paris, France, [5] Department of Sociology, University of Konstanz, Universitätsstraße 10, 78464 Konstanz, Germany.

[*] Corresponding author. Email: johanna.koechling@uni-konstanz.de

[+] These authors contributed equally: Johanna Köchling (JK), Julia E. Koller (JEK)

This PDF file includes:

Supplementary Figure 1. Predicted perceptions of ideal, actual and personal carbon footprints for participants within the five personal wealth quintiles.
Supplementary Figure 2. Perceptions of actual carbon footprint distributions across national wealth quintiles by personal wealth quintiles.
Supplementary Figure 3. Perceptions of personal carbon footprints by personal numeric wealth groups.
Supplementary Figure 4. Comparison between perceived and objective carbon footprint distributions by personal wealth quintile.
Supplementary Table 1. Perceptions of ideal carbon footprints
Supplementary Table 2. Perceptions of ideal carbon footprints controlled for gender
Supplementary Table 3. Perceptions of ideal carbon footprints controlled for household size
Supplementary Table 4. Perceptions of actual carbon footprints
Supplementary Table 5. Perceptions of actual carbon footprints controlled for gender
Supplementary Table 6. Perceptions of actual carbon footprints controlled for household size
Supplementary Table 7. Perceptions of personal carbon footprints
Supplementary Table 8. Multilevel model for perceptions of ideal, actual and personal carbon footprints among participants within the five personal wealth quintiles
Supplementary Table 9. Cross table for personal wealth quintiles and personal numeric wealth groups
Supplementary Methods. Original Items

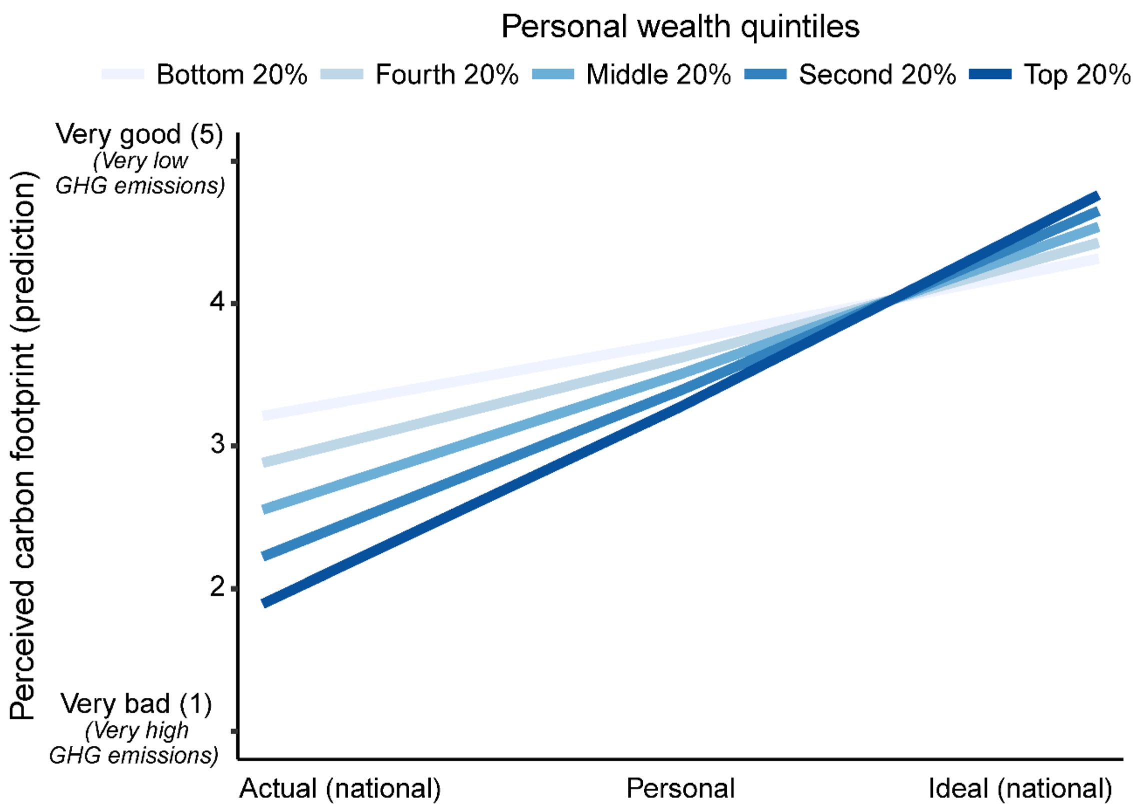

Supplementary Figure 1. **Predicted perceptions of ideal, actual and personal carbon footprints for participants within the five personal wealth quintiles.** The figure displays the results from a multilevel model (n = 1,384; see Supplementary Table 8). Participants from five personal wealth quintiles (blue colors) rated their own (personal) carbon footprint and the actual (national) and ideal (national) carbon footprints of others within their own wealth quintile. Wealthier participants viewed the actual (national) carbon footprint for their own wealth quintile as worse compared to less wealthy participants (b = -0.33, 95% CI[-0.38;-0.28], t(4083.60) = -14.05, p < .001). While wealthier participants evaluated their own carbon footprint as slightly worse (i.e., higher GHG emissions) than less wealthy participants (b = 0.21, 95% CI[0.15;0.28], t(2750.39) = 6.75 p < .001), they more strongly believed that their carbon footprint is better than that of others in their wealth quintile. Moreover, wealthier participants desired a comparably ambitious ideal carbon footprint for their wealth quintile (b = 0.44, 95% CI[0.38;0.50], t(2753.11) = 13.84, p < .001). For example, participants from the top 20% personal wealth quintile (dark blue line) rated their wealth quintiles' carbon footprint as the worst and set the highest ideal for their wealth quintile in society.

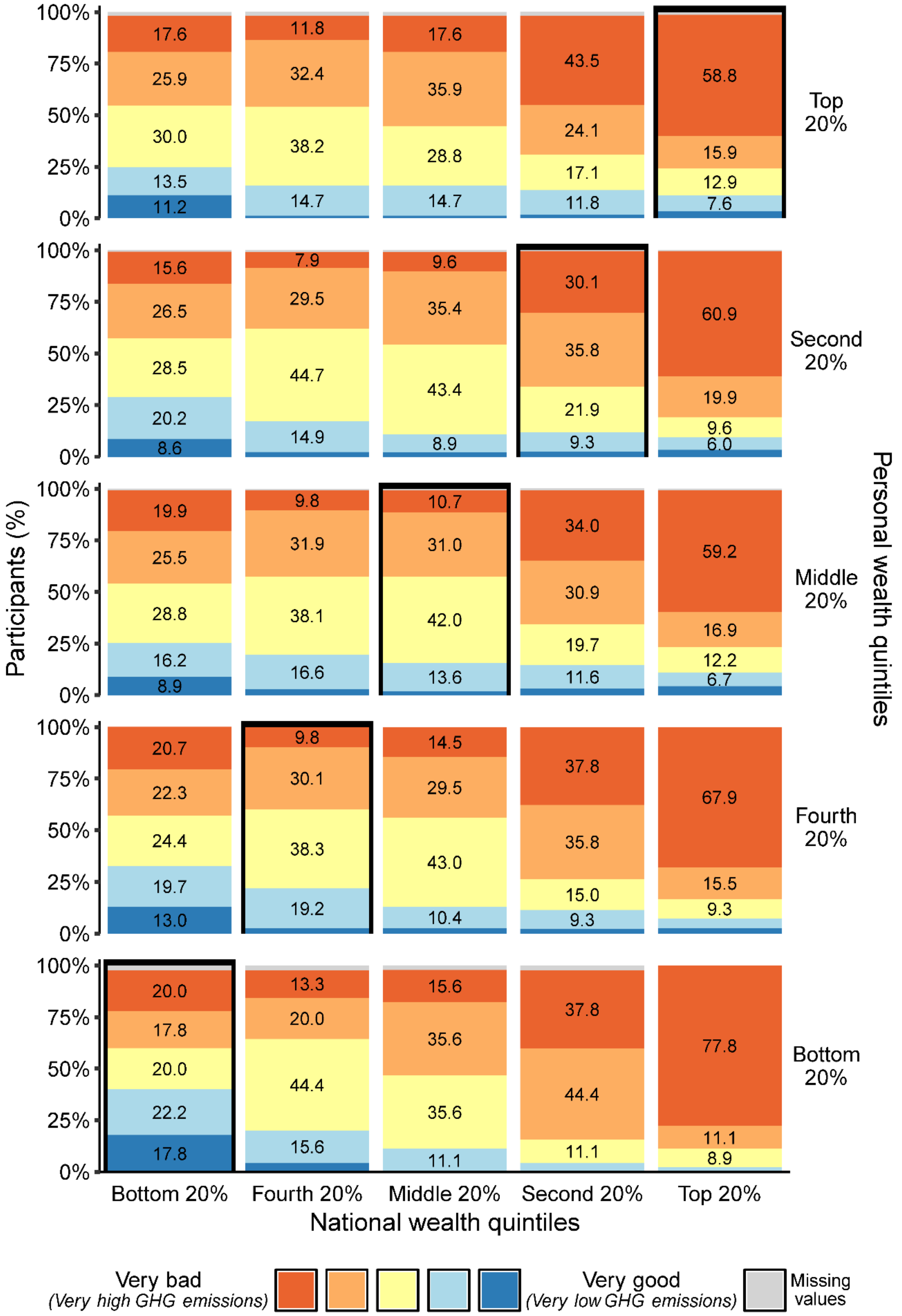

Supplementary Figure 2. **Perceptions of actual carbon footprint distributions across national wealth quintiles by personal wealth quintiles.** Displayed is the percentage of participants (%) who estimated the actual carbon footprint of five wealth quintiles as “very

bad" (red) to "very good" (dark blue) split by participants' personal wealth quintile. Black frames highlight ratings of others' carbon footprints within the participant's own wealth quintile (in-group ratings). For instance, 58.8% of individuals within the top 20% wealth quintile believe that average carbon footprints of the top 20% wealth quintile are currently "very bad" (i.e., very high GHG emissions; top right corner). Values below 5%, including missing values, are not labeled for presentation reasons. The number of participants per personal wealth quintile, with missing values in parenthesis, is as follows: top 20%: 170 (2-3), second 20%: 302 (1-2), middle 20%: 674 (5-6), fourth 20%: 193 (0), bottom 20%: 45 (0-1).

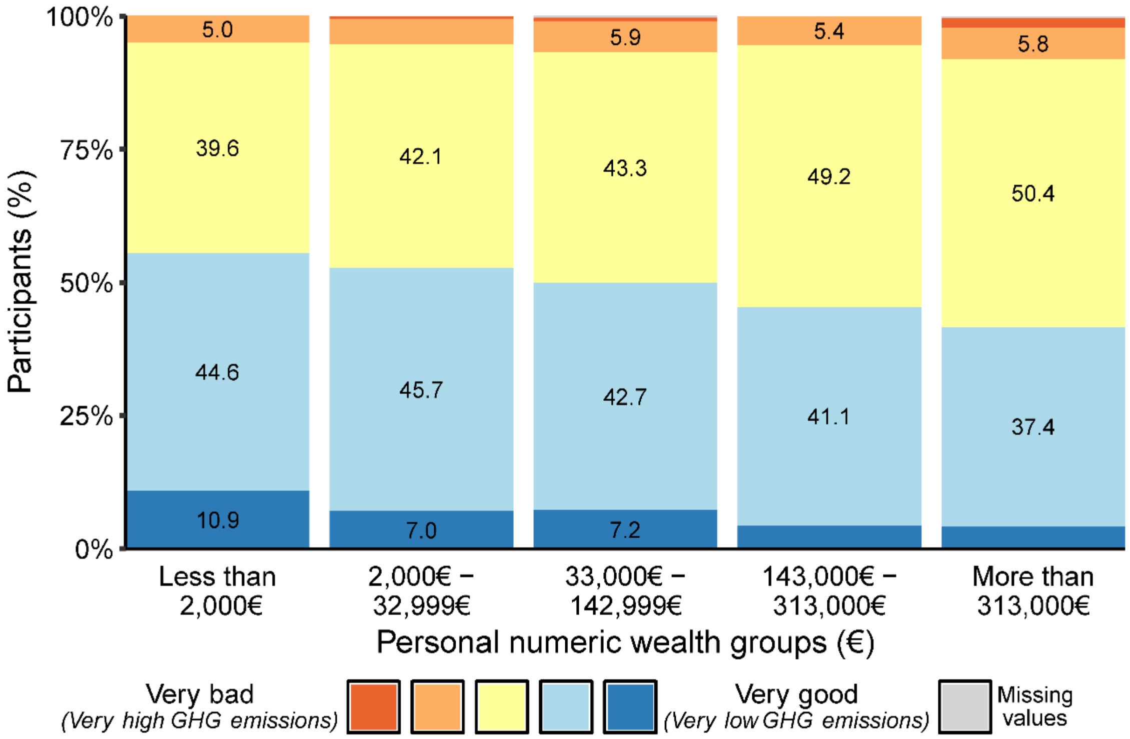

Supplementary Figure 3. **Perceptions of personal carbon footprints by personal numeric wealth groups.** Displayed the percentage (%) of participants who rated their personal carbon footprint as "very bad" (red) to "very good" (dark blue) by personal numeric wealth group (in euros, €). Wealthier groups perceived their own carbon footprint as worse (b = -0.06, 95% CI [-0.09;-0.03], $t(1353) = -4.02$, $R^2 = 0.01$, r = -.11, 95% CI [-.16;-.06], $p < .001$; Supplementary Table 7). Values below 5% (including missing values) are not labeled for presentation reasons. The number of participants per personal numeric wealth group is as follows: top 20%: 376, second 20%: 185, middle 20%: 306, fourth 20%: 387, bottom 20%: 101, missing values: 31.

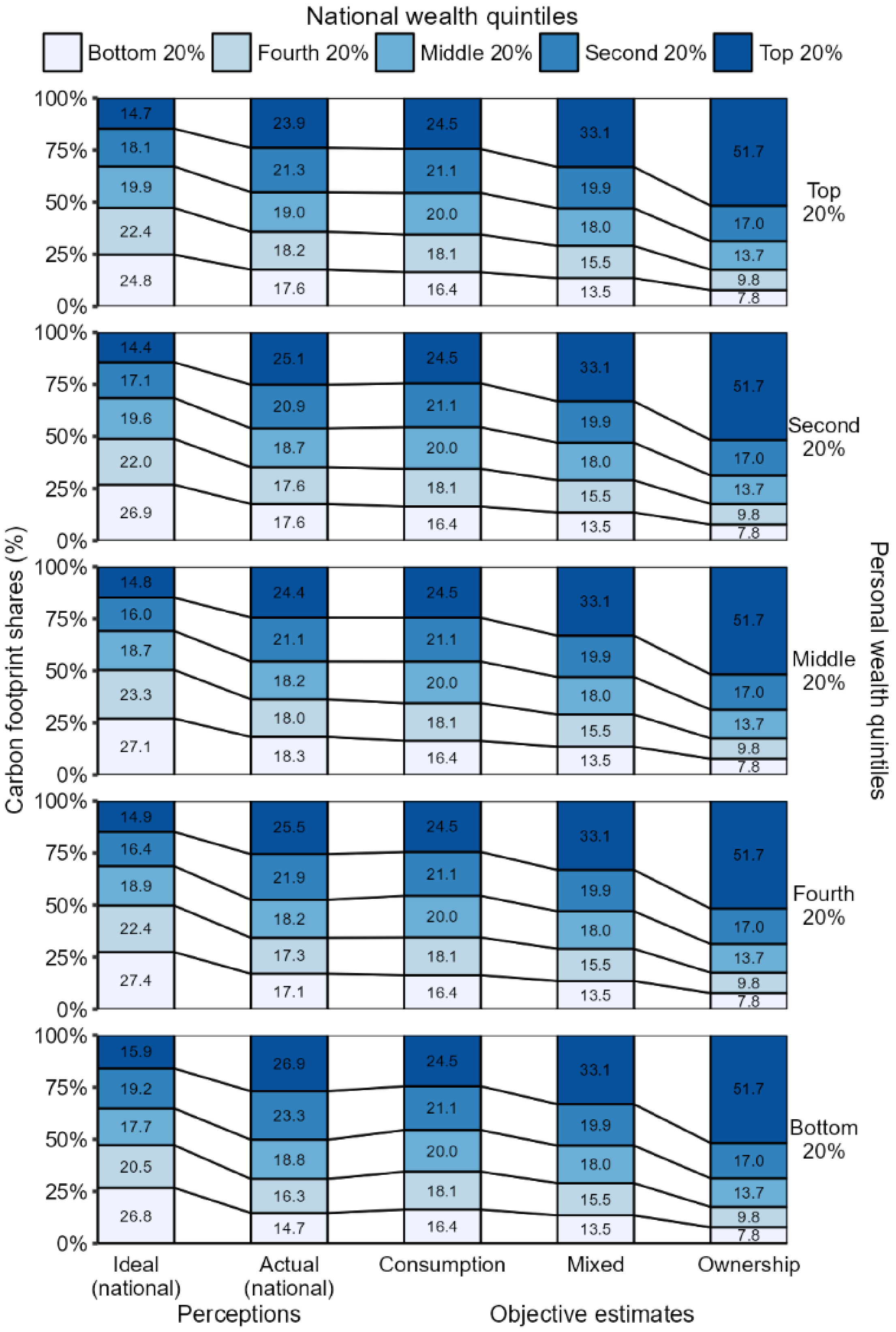

Supplementary Figure 4. **Comparison between perceived and objective carbon footprint distributions by personal wealth quintile.** Higher shares represent a worse carbon footprint of the respective national wealth quintile (bottom 20% light blue – top 20% dark blue). Ratings

of ideal and actual carbon footprints for each of the five wealth quintiles were reverse coded, divided by the total sum of these ratings, and converted into percentages (%). For the calculation of ideal and actual shares, participants with missings on one or more items used to calculate each respective share had to be excluded. The number of participants per personal wealth quintile included in the calculation of ideal/actual shares is as follows: top 20%: 167/ 167, second 20%: 301/ 300, middle 20%: 669/ 668, fourth 20%: 191/ 193, bottom 20%: 44/ 44.

Supplementary Table 1 Perceptions of ideal carbon footprints

	Random slopes model (fixed effects)					Random intercept model (fixed effects)				
Predictor	*b* [95% CI]	*SE*	*t*	*df*	*p*	*b* [95% CI]	*SE*	*t*	*df*	*p*
Model with national wealth quintile only (Pseudo-R^2 = 0.65)										
Intercept	4.52 [4.49;4.56]	0.018	251.33	1375.98	<.001	4.52 [4.49;4.56]	0.018	251.20	1376.41	<.001
National wealth quintile	0.10 [0.08;0.11]	0.006	15.13	1375.58	<.001	0.10 [0.09;0.10]	0.004	25.43	5501.60	<.001
Model with personal wealth quintile (Pseudo-R^2 = 0.65)										
Intercept	4.51 [4.47;4.55]	0.019	242.12	1374.55	<.001	4.51 [4.47;4.55]	0.019	242.00	1374.83	<.001
National wealth quintile	0.10 [0.09;0.11]	0.007	15.14	1373.96	<.001	0.10 [0.09;0.11]	0.004	25.46	5494.34	<.001
Personal wealth quintile	0.05 [0.01;0.09]	0.019	2.73	1377.44	.006	0.05 [0.01;0.09]	0.019	2.75	1377.11	.006
National x personal wealth quintile	-0.01 [-0.03;0.00]	0.007	-2.09	1375.56	.037	-0.01 [-0.02;-0.01]	0.004	-3.58	5498.84	<.001
Model with personal numeric wealth group (€; Pseudo-R^2 = 0.64)										
Intercept	4.52 [4.49;4.56]	0.018	247.23	1347.23	<.001	4.52 [4.49;4.56]	0.018	247.11	1347.62	<.001
National wealth quintile	0.10 [0.09;0.12]	0.007	15.64	1346.44	<.001	0.10 [0.09;0.11]	0.004	26.19	5387.92	<.001
Personal wealth group	0.01 [-0.02;0.03]	0.014	0.47	1347.59	.638	0.01 [-0.02;0.03]	0.014	0.47	1347.89	.638
National x personal wealth group	-0.01 [-0.02;0.00]	0.005	-2.48	1346.67	.013	-0.01 [-0.02;-0.01]	0.003	-4.15	5388.49	<.001

Note. ICC = 0.64. Results from multilevel analyses (two-sided). The random slopes models were preferred. Pseudo-R^2 is reported for the random slopes models. The predictors national wealth quintile and personal wealth group (quintile and numeric) were recoded so that the intercept refers to the ideal carbon footprint predicted for the middle national wealth quintile and middle personal wealth group (quintile and numeric). Coefficients are unstandardized.

Supplementary Table 2 Perceptions of ideal carbon footprints controlled for gender

	Random slopes model (fixed effects)					Random intercept model (fixed effects)				
Predictor	*b [95% CI]*	*SE*	*t*	*df*	*p*	*b [95% CI]*	*SE*	*t*	*df*	*p*
Model with national wealth quintile only (Pseudo-R^2 = 0.65)										
Intercept	4.54 [4.50;4.59]	0.023	199.47	1391.92	<.001	4.54 [4.50;4.59]	0.023	198.09	1369.94	<.001
National wealth quintile	0.10 [0.08;0.11]	0.006	15.03	1368.54	<.001	0.10 [0.09;0.10]	0.004	25.27	5473.56	<.001
Gender	-0.06 [-0.13;0.01]	0.037	-1.63	1369.15	.103	-0.06 [-0.13;0.01]	0.037	-1.61	1369.01	.108
Model with personal wealth quintile, controlling for gender (Pseudo-R^2 = 0.65)										
Intercept	4.54 [4.49;4.58]	0.023	198.08	1388.19	<.001	4.54 [4.49;4.58]	0.023	196.93	1368.14	<.001
National wealth quintile	0.10 [0.09;0.11]	0.007	15.05	1366.97	<.001	0.10 [0.09;0.11]	0.004	25.31	5466.33	<.001
Personal wealth quintile	0.06 [0.02;0.10]	0.019	3.10	1372.92	.002	0.06 [0.02;0.10]	0.019	3.13	1370.14	.002
National x personal wealth quintile	-0.01 [-0.03;0.00]	0.007	-2.11	1368.56	.035	-0.01 [-0.02;-0.01]	0.004	-3.60	5470.81	<.001
Gender	-0.08 [-0.15;-0.01]	0.037	-2.10	1367.38	.036	-0.08 [-0.15;-0.01]	0.038	-2.14	1367.19	.032
Model with personal numeric wealth group (€), controlling for gender (Pseudo-R^2 = 0.65)										
Intercept	4.54 [4.50;4.59]	0.023	199.29	1373.82	<.001	4.54 [4.50;4.59]	0.023	197.70	1346.96	<.001
National wealth quintile	0.10 [0.09;0.12]	0.007	15.61	1345.43	<.001	0.10 [0.09;0.11]	0.004	26.14	5383.86	<.001
Personal wealth group	0.01 [-0.02;0.03]	0.014	0.56	1346.78	.573	0.01 [-0.02;0.03]	0.014	0.57	1346.75	.569
National x personal wealth group	-0.01 [-0.02;0.00]	0.005	-2.50	1345.65	.013	-0.01 [-0.02;-0.01]	0.003	-4.19	5384.44	<.001
Gender	-0.05 [-0.12;0.02]	0.036	-1.40	1345.95	.162	-0.05 [-0.13;0.02]	0.037	-1.47	1345.76	.142

Note. ICC = 0.64. Results from multilevel analyses (two-sided). The random slopes models were preferred. Pseudo-R^2 is reported for the random slopes models. The predictors national wealth quintile and personal wealth group (quintile and numeric) were recoded so that the intercept refers to the ideal carbon footprint predicted for the middle national wealth quintile and middle personal wealth group (quintile and numeric). For gender, zero refers to women. Coefficients are unstandardized.

Supplementary Table 3 Perceptions of ideal carbon footprints controlled for household size

	Random slopes model (fixed effects)					Random intercept model (fixed effects)				
Predictor	*b [95% CI]*	*SE*	*t*	*df*	*p*	*b [95% CI]*	*SE*	*t*	*df*	*p*
Model with national wealth quintile only, controlling for household size (Pseudo-R2 = 0.65)										
Intercept	4.52 [4.47;4.57]	0.024	187.58	1400.89	<.001	4.52 [4.47;4.57]	0.024	185.93	1377.18	<.001
National wealth quintile	0.10 [0.08;0.11]	0.006	15.13	1375.56	<.001	0.10 [0.09;0.10]	0.004	25.43	5501.61	<.001
Household size	0.00 [-0.03;0.03]	0.016	0.05	1376.38	.959	0.00 [-0.03;0.03]	0.016	0.22	1376.25	.823
Model with personal wealth quintile, controlling for household size (Pseudo-R^2 = 0.65)										
Intercept	4.52 [4.47;4.56]	0.024	187.26	1397.60	<.001	4.52 [4.47;4.56]	0.024	185.85	1375.30	<.001
National wealth quintile	0.10 [0.09;0.11]	0.007	15.14	1373.96	<.001	0.10 [0.09;0.11]	0.004	25.46	5494.33	<.001
Personal wealth quintile	0.05 [0.02;0.09]	0.019	2.78	1382.97	.005	0.05 [0.02;0.09]	0.019	2.78	1377.27	.006
National x personal wealth quintile	-0.01 [-0.03;0.00]	0.007	-2.09	1375.55	.037	-0.01 [-0.02;-0.01]	0.004	-3.58	5498.83	<.001
Household size	-0.01 [-0.04;0.02]	0.016	-0.55	1374.66	.586	-0.01 [-0.04;0.02]	0.016	-0.46	1374.52	.643
Model with personal numeric wealth group (€), controlling for household size (Pseudo-R^2 = 0.64)										
Intercept	4.53 [4.48;4.57]	0.024	187.84	1376.65	<.001	4.52 [4.48;4.57]	0.024	185.96	1347.79	<.001
National wealth quintile	0.10 [0.09;0.12]	0.007	15.64	1346.44	<.001	0.10 [0.09;0.11]	0.004	26.19	5387.91	<.001
Personal wealth group	0.01 [-0.02;0.03]	0.014	0.49	1355.49	.626	0.01 [-0.02;0.03]	0.014	0.47	1347.51	.636
National x personal wealth group	-0.01 [-0.02;0.00]	0.005	-2.48	1346.66	.013	-0.01 [-0.02;-0.01]	0.003	-4.15	5388.49	<.001
Household size	0.00 [-0.03;0.03]	0.016	-0.13	1346.84	.897	0.00 [-0.03;0.03]	0.017	-0.08	1346.70	.939

Note. ICC = 0.64. Results from multilevel analyses (two-sided). The random slopes models were preferred. Pseudo-R^2 is reported for the random slopes models. The predictors national wealth quintile and personal wealth group (quintile and numeric) were recoded so that the intercept refers to the ideal carbon footprint predicted for the middle national wealth quintile and middle personal wealth group (quintile and numeric). For household size, zero refers to single households. Coefficients are unstandardized.

Supplementary Table 4 Perceptions of actual carbon footprints

	Random slopes model (fixed effects)					Random intercept model (fixed effects)				
Predictor	*b [95% CI]*	*SE*	*t*	*df*	*p*	*b [95% CI]*	*SE*	*t*	*df*	*p*
Model with national wealth quintile only (Pseudo-R2 = 0.61)										
Intercept	2.38 [2.34;2.42]	0.019	126.30	1377.58	<.001	2.38 [2.34;2.42]	0.019	126.35	1377.42	<.001
National wealth quintile	-0.26 [-0.28;-0.24]	0.013	-20.81	1378.28	<.001	-0.26 [-0.28;-0.25]	0.008	-33.52	5504.36	<.001
Model with personal wealth quintile (Pseudo-R^2 = 0.61)										
Intercept	2.38 [2.34;2.42]	0.020	121.94	1375.62	<.001	2.38 [2.34;2.42]	0.020	121.99	1375.49	<.001
National wealth quintile	-0.27 [-0.29;-0.24]	0.013	-20.59	1376.34	<.001	-0.27 [-0.28;-0.25]	0.008	-33.15	5496.41	<.001
Personal wealth quintile	0.00 [-0.04;0.04]	0.020	0.09	1378.39	.928	0.00 [-0.04;0.04]	0.020	0.08	1378.39	.939
National x personal wealth quintile	0.02 [0.00;0.05]	0.013	1.60	1379.30	.110	0.02 [0.01;0.04]	0.008	2.62	5502.44	.009
Model with personal numeric wealth group (€; Pseudo-R^2 = 0.61)										
Intercept	2.38 [2.34;2.42]	0.019	122.99	1349.69	<.001	2.38 [2.34;2.42]	0.019	123.04	1349.46	<.001
National wealth quintile	-0.27 [-0.29;-0.24]	0.013	-20.90	1350.39	<.001	-0.27 [-0.28;-0.25]	0.008	-33.65	5392.53	<.001
Personal wealth group	0.00 [-0.03;0.03]	0.014	0.04	1350.71	.968	0.00 [-0.03;0.03]	0.014	0.02	1350.52	.988
National x personal wealth group	0.04 [0.02;0.06]	0.010	3.85	1351.37	<.001	0.04 [0.03;0.05]	0.006	6.22	5394.93	<.001

Note. ICC = 0.22. Results from multilevel analyses (two-sided). The random slopes models were preferred. Pseudo-R^2 is reported for the random slopes models. The predictors national wealth quintile and personal wealth group (quintile and numeric) were recoded so that the intercept refers to the actual carbon footprint predicted for the middle national wealth quintile and middle personal wealth group (quintile and numeric). Coefficients are unstandardized.

Supplementary Table 5 Perceptions of actual carbon footprints controlled for gender

	Random slopes model (fixed effects)					Random intercept model (fixed effects)				
Predictor	*b [95% CI]*	*SE*	*t*	*df*	*p*	*b [95% CI]*	*SE*	*t*	*df*	*p*
Model with national wealth quintile only, controlling for gender (Pseudo-R2 = 0.61)										
Intercept	2.43 [2.39;2.48]	0.024	101.89	1373.04	<.001	2.43 [2.39;2.48]	0.024	101.90	1371.43	<.001
National wealth quintile	-0.26 [-0.29;-0.24]	0.013	-20.77	1371.28	<.001	-0.26 [-0.28;-0.25]	0.008	-33.47	5476.42	<.001
Gender	-0.14 [-0.22;-0.06]	0.039	-3.63	1369.90	<.001	-0.14 [-0.22;-0.07]	0.039	-3.65	1369.86	<.001
Model with personal wealth quintile, controlling for gender (Pseudo-R^2 = 0.61)										
Intercept	2.43 [2.38;2.48]	0.024	101.19	1370.69	<.001	2.43 [2.38;2.48]	0.024	101.21	1369.24	<.001
National wealth quintile	-0.27 [-0.29;-0.24]	0.013	-20.52	1369.32	<.001	-0.27 [-0.28;-0.25]	0.008	-33.06	5468.45	<.001
Personal wealth quintile	0.01 [-0.03;0.05]	0.020	0.66	1371.71	.510	0.01 [-0.03;0.05]	0.020	0.65	1371.58	.515
National x personal wealth quintile	0.02 [-0.01;0.05]	0.013	1.53	1372.27	.126	0.02 [0.00;0.04]	0.008	2.51	5474.44	.012
Gender	-0.14 [-0.22;-0.06]	0.039	-3.60	1368.01	<.001	-0.14 [-0.22;-0.07]	0.039	-3.63	1367.90	<.001
Model with personal numeric wealth group (€), controlling for gender (Pseudo-R^2 = 0.61)										
Intercept	2.43 [2.38;2.48]	0.024	100.40	1351.30	<.001	2.43 [2.38;2.48]	0.024	100.41	1349.36	<.001
National wealth quintile	-0.27 [-0.29;-0.24]	0.013	-20.95	1349.41	<.001	-0.27 [-0.29;-0.25]	0.008	-33.71	5388.59	<.001
Personal wealth group	0.00 [-0.02;0.03]	0.014	0.23	1349.73	.816	0.00 [-0.02;0.03]	0.014	0.21	1349.53	.833
National x personal wealth group	0.04 [0.02;0.05]	0.010	3.83	1350.39	<.001	0.04 [0.02;0.05]	0.006	6.18	5390.98	<.001
Gender	-0.13 [-0.21;-0.06]	0.039	-3.40	1347.79	<.001	-0.13 [-0.21;-0.06]	0.039	-3.44	1347.62	<.001

Note. ICC = 0.22. Results from multilevel analyses (two-sided). The random slopes models were preferred. Pseudo-R^2 is reported for the random slopes models. The predictors national wealth quintile and personal wealth group (quintile and numeric) were recoded so that the intercept refers to the actual carbon footprint predicted for the middle national wealth quintile and middle personal wealth group (quintile and numeric). For gender, zero refers to women. Coefficients are unstandardized.

Supplementary Table 6 Perceptions of actual carbon footprints controlled for household size

	Random slopes model (fixed effects)					Random intercept model (fixed effects)				
Predictor	*b [95% CI]*	*SE*	*t*	*df*	*p*	*b [95% CI]*	*SE*	*t*	*df*	*p*
Model with national wealth quintile only, controlling for household size (Pseudo-R2 = 0.61)										
Intercept	2.36 [2.31;2.41]	0.025	92.72	1379.80	<.001	2.36 [2.31;2.41]	0.025	92.63	1378.12	<.001
National wealth quintile	-0.26 [-0.28;-0.24]	0.013	-20.81	1378.28	<.001	-0.26 [-0.28;-0.25]	0.008	-33.52	5504.32	<.001
Household size	0.02 [-0.01;0.05]	0.017	1.09	1376.73	.275	0.02 [-0.01;0.05]	0.017	1.21	1376.74	.226
Model with personal wealth quintile, controlling for household size (Pseudo-R^2 = 0.61)										
Intercept	2.36 [2.31;2.41]	0.026	92.63	1377.62	<.001	2.36 [2.31;2.41]	0.026	92.56	1376.01	<.001
National wealth quintile	-0.27 [-0.29;-0.24]	0.013	-20.58	1376.32	<.001	-0.27 [-0.28;-0.25]	0.008	-33.15	5496.39	<.001
Personal wealth quintile	0.00 [-0.04;0.04]	0.020	-0.19	1378.92	.848	0.00 [-0.04;0.04]	0.020	-0.23	1378.60	.817
National x personal wealth quintile	0.02 [0.00;0.05]	0.013	1.60	1379.28	.109	0.02 [0.01;0.04]	0.008	2.63	5502.41	.009
Household size	0.02 [-0.01;0.05]	0.017	1.11	1374.90	.265	0.02 [-0.01;0.05]	0.017	1.22	1374.88	.223
Model with personal numeric wealth group (€), controlling for household size (Pseudo-R^2 = 0.61)										
Intercept	2.36 [2.31;2.41]	0.026	91.77	1352.07	<.001	2.36 [2.31;2.41]	0.026	91.69	1349.85	<.001
National wealth quintile	-0.27 [-0.29;-0.24]	0.013	-20.90	1350.38	<.001	-0.27 [-0.28;-0.25]	0.008	-33.65	5392.49	<.001
Personal wealth group	0.00 [-0.03;0.02]	0.015	-0.29	1351.02	.771	-0.01 [-0.03;0.02]	0.015	-0.34	1350.20	.735
National x personal wealth group	0.04 [0.02;0.06]	0.010	3.85	1351.36	<.001	0.04 [0.03;0.05]	0.006	6.22	5394.89	<.001
Household size	0.02 [-0.01;0.05]	0.017	1.17	1348.36	.244	0.02 [-0.01;0.06]	0.018	1.25	1348.26	.213

Note. ICC = 0.22. Results from multilevel analyses (two-sided). The random slopes models were preferred. Pseudo-R^2 is reported for the random slopes models. The predictors national wealth quintile and personal wealth group (quintile and numeric) were recoded so that the intercept refers to the actual carbon footprint predicted for the middle national wealth quintile and middle personal wealth group (quintile and numeric). For household size, zero refers to single households. Coefficients are unstandardized.

Supplementary Table 7 Perceptions of personal carbon footprints

Predictor	*b [95% CI]*	SE	*t*	*p*
Model with personal wealth quintile (R^2 = 0.02, r = -.15, 95% CI [-.20;-.10], df = 1380)				
Intercept	3.85 [3.72;3.99]	0.069	55.98	<.001
Personal wealth quintile	-0.11 [-0.15;-0.08]	0.020	-5.67	<.001
Model with personal numeric wealth group (€; R^2 = 0.01, r = -.11, 95% CI [-.16;-.06], df = 1353)				
Intercept	3.67 [3.57;3.77]	0.05	70.42	<.001
Personal wealth group	-0.06 [-0.09;-0.03]	0.01	-4.02	<.001
Model with personal wealth quintile controlling for gender (R^2 = 0.03, df = 1372)				
Intercept	3.86 [3.73;4.00]	0.069	55.97	<.001
Personal wealth quintile	-0.11 [-0.15;-0.07]	0.021	-5.13	<.001
Gender	-0.11 [-0.19;-0.03]	0.041	-2.68	.008
Model with personal wealth quintile controlling for household size (R^2 = 0.02, df = 1379)				
Intercept	3.85 [3.72;3.99]	0.069	56.02	<.001
Personal wealth quintile	-0.11 [-0.15;-0.07]	0.021	-5.11	<.001
Household size	-0.03 [-0.06;0.01]	0.018	-1.55	.121
Model with personal numeric wealth group (€) controlling for gender (R^2 = 0.02, df = 1351)				
Intercept	3.71 [3.60;3.81]	0.053	69.48	<.001
Personal wealth group	-0.06 [-0.09;-0.03]	0.014	-3.80	<.001
Gender	-0.13 [-0.21;-0.05]	0.040	-3.21	.001
Model with personal numeric wealth group (€) controlling for household size (R^2 = 0.01, df = 1352)				
Intercept	3.68 [3.57;3.78]	0.052	70.42	<.001
Personal wealth group	-0.05 [-0.08;-0.02]	0.015	-3.28	.001
Household size	-0.04 [-0.07;0.00]	0.018	-2.00	.046

Note. Results from linear regression analyses (two-sided). The predictor personal wealth group (quintile and numeric) was recoded so that the intercept refers to the personal carbon footprint predicted for the middle personal wealth group (quintile and numeric). For gender, zero refers to women and for household size, zero refers to single households. Coefficients are unstandardized.

Supplementary Table 8 Multilevel model for perceptions of ideal, actual and personal carbon footprints among participants within the five personal wealth quintiles

Predictor	Random intercept model (fixed effects)				
	b [95% CI]	SE	*t*	df	*p*
Intercept	2.55 [2.51;2.60]	0.023	110.13	4083.20	<.001
Personal (ref.: actual)	0.95 [0.89;1.02]	0.031	30.32	2748.68	<.001
Ideal (ref.: actual)	1.99 [1.93;2.05]	0.031	63.14	2750.33	<.001
Personal wealth quintile	-0.33 [-0.38;-0.28]	0.023	-14.05	4083.60	<.001
Personal x personal wealth quintile	0.21 [0.15;0.28]	0.032	6.75	2750.39	<.001
Ideal x personal wealth quintile	0.44 [0.38;0.50]	0.032	13.84	2753.11	<.001

Note. ICC = 0.00. Pseudo-R^2 = 0.57. Results from multilevel analyses (two-sided). The random slopes model was unidentifiable. The predictor personal wealth quintile was recoded so that the intercept refers to the perceived carbon footprint predicted for participants in the middle personal wealth quintile. Coefficients are unstandardized.

Supplementary Table 9 Cross table for personal wealth quintiles and personal numeric wealth groups

Personal numeric wealth group (€)	Personal wealth quintile					
	Bottom 20%	Fourth 20%	Middle 20%	Second 20%	Top 20%	Missing values
Less than 2,000€	17 (1.2%)	39 (2.8%)	36 (2.6%)	4 (0.3%)	5 (0.4%)	0 (0.0%)
2,000 to 32,999€	23 (1.7%)	96 (6.9%)	216 (15.6%)	46 (3.3%)	6 (0.4%)	0 (0.0%)
33,000 to 142,999€	4 (0.3%)	29 (2.1%)	177 (12.8%)	69 (5.0%)	27 (1.9%)	1 (0.1%)
143,000 to 313,000€	1 (0.1%)	15 (1.1%)	89 (6.4%)	55 (4.0%)	25 (1.8%)	0 (0.0%)
More than 313,000€	0 (0.0%)	7 (0.5%)	145 (10.5%)	122 (8.8%)	103 (7.4%)	0 (0.0%)
Missing values	0 (0.0%)	7 (0.5%)	11 (0.8%)	6 (0.4%)	4 (0.3%)	1 (0.1%)

Note. Participants self-categorized themselves to one of five wealth quintiles and numeric wealth groups (€)

Supplementary Methods. Original Items [with English translations]

1. Importance of a good carbon footprint.

Wie wichtig ist Ihnen Folgendes?

Für mich ist eine ...

	sehr unwichtig	eher unwichtig	weder noch	eher wichtig	sehr wichtig
... gute Klimabilanz ... (d.h. geringe Treibhausgasemissionen)	❑$_1$	❑$_2$	❑$_3$	❑$_4$	❑$_5$

[How Important is the following to you? For me, a good carbon footprint (i.e., low greenhouse gas emissions) is…
very unimportant (1)/ rather unimportant (2)/ neither important nor important (3)/ rather important (4)/ very important (5)]

2. Personal carbon footprint.

Wie ist Ihre momentane Klimabilanz (Treibhausgasemissionen)?

sehr schlecht	schlecht	mittelmäßig	gut	sehr gut
❑$_1$	❑$_2$	❑$_3$	❑$_4$	❑$_5$

[How would you rate your current carbon footprint (greenhouse gas emissions)?
Very bad (1)/ bad (2)/ average (3)/ good (4)/ very good (5)]

3. Personal wealth group.

Ich würde mich auf Grund meiner momentanen finanziellen Situation der folgenden Gruppe zuordnen:

Untere 20% der Bevölkerung	❑$_1$
Zweite 20% der Bevölkerung	❑$_2$
Mittlere 20% der Bevölkerung	❑$_3$
Vierte 20% der Bevölkerung	❑$_4$
Obere 20% der Bevölkerung	❑$_5$

[Based on my current financial situation, I would consider myself as belonging to the following group:
bottom 20% of the population/ second 20% of the population/ middle 20% of the population/ fourth 20% of the population/ top 20% of the population]

4. Perception of ideal carbon footprint distributions in society.

Wie klimafreundlich ist Deutschland?

Das Vermögen in einem Land ist unterschiedlich auf die Bevölkerung verteilt. Geordnet von den Reichsten bis zu den Ärmsten, teilen wir die Bevölkerung Deutschlands in 5 Stufen ein, wobei jede Stufe 20% der Haushalte umfasst.

Das Vermögen eines Haushaltes wird als der Gesamtwert des Besitzes abzüglich der Schulden definiert. Es beinhaltet Erspartes zuzüglich des Wertes anderer Dinge wie Eigentum, Aktien, Wertpapiere, Kunst, Sammlungen usw. abzüglich des Wertes von Dingen wie Kredite und Hypotheken.

Schätzen Sie nun, wie die Klima-Bilanz (Treibhausgasemissionen) von Personen in den folgenden 5 Vermögensstufen durchschnittlich ist.

1. Vermögens-Stufe: Obere 20%

sehr schlecht … sehr gut

2. Vermögens-Stufe: Zweite 20%

sehr schlecht … sehr gut

3. Vermögens-Stufe: Mittlere 20%

sehr schlecht … sehr gut

4. Vermögens-Stufe: Vierte 20%

sehr schlecht … sehr gut

5. Vermögens-Stufe: Untere 20%

sehr schlecht … sehr gut

[How climate-friendly is Germany?
Wealth is distributed differently among the population within a country. Ranked from the richest to the poorest, we divide the German Population into five groups, each containing 20% of households. The wealth of a household is defined as the total value of everything owned minus any debt. It includes savings plus the value of other things such as property, stocks, bonds, art, collections, etc., minus the value of things like loans and mortgages

How would you rate the average current carbon footprint (greenhouse gas emissions) of people in the following five wealth groups.

1. Wealth group: top 20% very bad … very good
2. Wealth group: second 20% very bad … very good
3. Wealth group: middle 20% very bad … very good
4. Wealth group: fourth 20% very bad … very good
5. Wealth group: bottom 20% very bad … very good]

5. Perception of actual carbon footprint distributions in society.

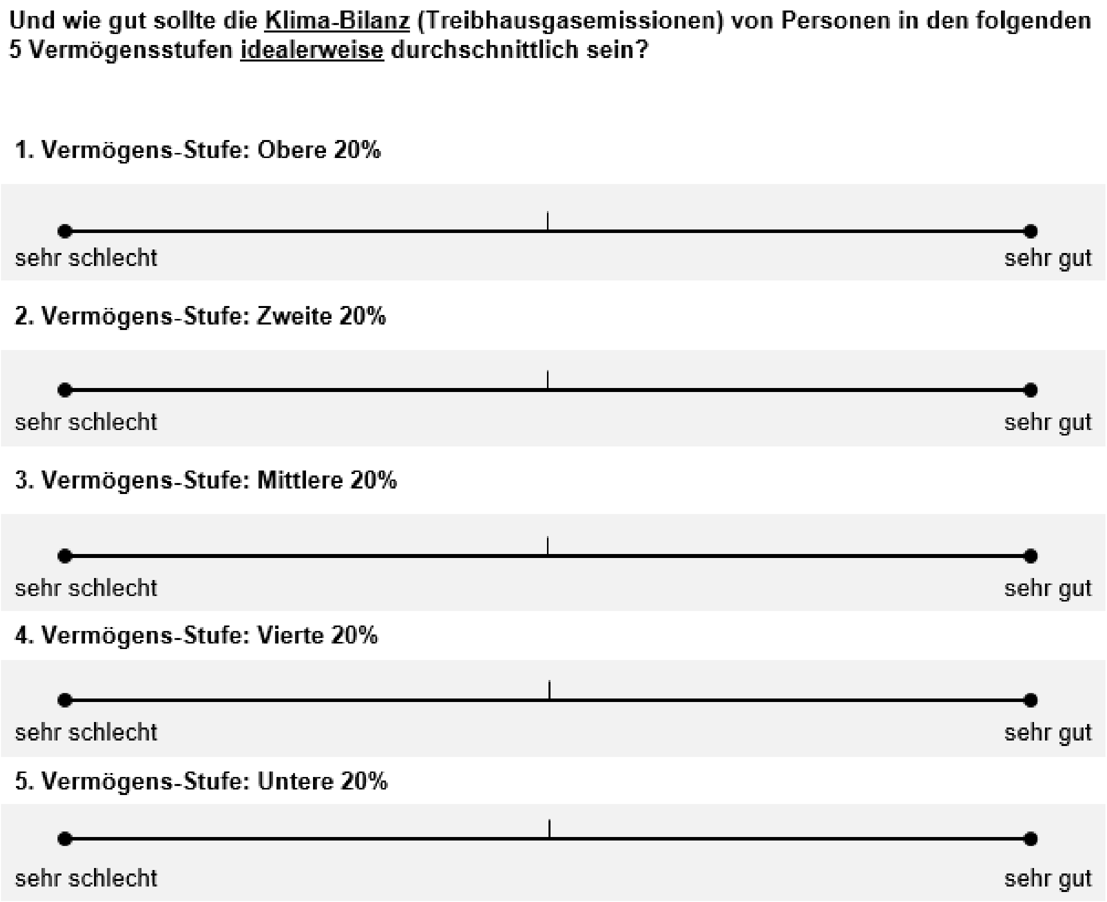
Und wie gut sollte die Klima-Bilanz (Treibhausgasemissionen) von Personen in den folgenden 5 Vermögensstufen idealerweise durchschnittlich sein?

1. Vermögens-Stufe: Obere 20%

sehr schlecht … sehr gut

2. Vermögens-Stufe: Zweite 20%

sehr schlecht … sehr gut

3. Vermögens-Stufe: Mittlere 20%

sehr schlecht … sehr gut

4. Vermögens-Stufe: Vierte 20%

sehr schlecht … sehr gut

5. Vermögens-Stufe: Untere 20%

sehr schlecht … sehr gut

[And how good should the average carbon footprint (greenhouse gas emissions) of people in the following five wealth groups ideally be.

1. Wealth group: top 20%	very bad … very good
2. Wealth group: second 20%	very bad … very good
3. Wealth group: middle 20%	very bad … very good
4. Wealth group: fourth 20%	very bad … very good
5. Wealth group: bottom 20%	very bad … very good]
