# Supplementary material for: The carbon perception gap in actual and ideal carbon footprints across wealth groups
Source: Nat Commun. 2025 Jul 4;16:6180. doi: 10.1038/s41467-025-61505-7 (PMC12227583; doi:10.1038/s41467-025-61505-7)
Supplement: Supplementary file 2 — Reporting Summary [file 41467_2025_61505_MOESM2_ESM.pdf]

nature portfolio

Corresponding author(s): Johanna Köchling

Last updated by author(s): May 26, 2025

Reporting Summary

Nature Portfolio wishes to improve the reproducibility of the work that we publish. This form provides structure for consistency and transparency in reporting. For further information on Nature Portfolio policies, see our Editorial Policies and the Editorial Policy Checklist.

Statistics

For all statistical analyses, confirm that the following items are present in the figure legend, table legend, main text, or Methods section.

n/a	Confirmed	
☐	☒	The exact sample size (*n*) for each experimental group/condition, given as a discrete number and unit of measurement
☐	☒	A statement on whether measurements were taken from distinct samples or whether the same sample was measured repeatedly
☐	☒	The statistical test(s) used AND whether they are one- or two-sided<br>*Only common tests should be described solely by name; describe more complex techniques in the Methods section.*
☒	☐	A description of all covariates tested
☐	☒	A description of any assumptions or corrections, such as tests of normality and adjustment for multiple comparisons
☐	☒	A full description of the statistical parameters including central tendency (e.g. means) or other basic estimates (e.g. regression coefficient) AND variation (e.g. standard deviation) or associated estimates of uncertainty (e.g. confidence intervals)
☐	☒	For null hypothesis testing, the test statistic (e.g. F, t, r) with confidence intervals, effect sizes, degrees of freedom and P value noted<br>*Give P values as exact values whenever suitable.*
☒	☐	For Bayesian analysis, information on the choice of priors and Markov chain Monte Carlo settings
☐	☒	For hierarchical and complex designs, identification of the appropriate level for tests and full reporting of outcomes
☐	☒	Estimates of effect sizes (e.g. Cohen's *d*, Pearson's *r*), indicating how they were calculated

Our web collection on statistics for biologists contains articles on many of the points above.

Software and code

Policy information about availability of computer code

Data collection	Unipark (EFS Spring 2023)
Data analysis	All statistical analyses were performed using R (version 4.4.2) and RStudio (version 2023.6.2.561). Data were structured using the R packages “data.table” (version 1.16.4), “dplyr” (version 1.1.4), “forcats” (version 1.0.0), and “tidyr” (version 1.3.1). For data visualizations, the packages “ggplot2” (version 3.5.2), “ggpubr” (version 0.6.0), “RColorBrewer” (version 1.1-3), “ggalluvial” (version 0.12.5), and “ggtext” (version 0.1.2) were used. Descriptive analyses were conducted with base R and the “Rmisc” package (version 1.5.1). Multilevel models were conducted using “lme4”(version 1.1-37) and “lmerTest” (version 3.1-3).

For manuscripts utilizing custom algorithms or software that are central to the research but not yet described in published literature, software must be made available to editors and reviewers. We strongly encourage code deposition in a community repository (e.g. GitHub). See the Nature Portfolio guidelines for submitting code & software for further information.

Data

Policy information about availability of data

All manuscripts must include a data availability statement. This statement should provide the following information, where applicable:

- Accession codes, unique identifiers, or web links for publicly available datasets
- A description of any restrictions on data availability
- For clinical datasets or third party data, please ensure that the statement adheres to our policy

The deidentified participant data used in this study are available in the publicly accessible repository KonData under a CC BY 4.0 license at https://doi.org/10.48606/WobQpECnfNuCRarU
The analysis code of this study is available in the publicly accessible repository KonData under a CC BY 4.0 license at https://doi.org/10.48606/WobQpECnfNuCRarU

Research involving human participants, their data, or biological material

Policy information about studies with human participants or human data. See also policy information about sex, gender (identity/presentation), and sexual orientation and race, ethnicity and racism.

Reporting on sex and gender	Participants were asked to self-report their gender as female, male or diverse. We did not ask for the biological "sex" as this is not common in German surveys. As we did not expect gender differences, we did not run any gender-based analyses. The disaggregated data for gender is available.
Reporting on race, ethnicity, or other socially relevant groupings	We did not collect data on the ethnicity/ race of participants. In Germany ethnic origin, race or related concepts are considered extremely sensitive data and are commonly not assessed. Since the end of the second world war, population statistics and socioeconomic data have generally not been collected on an ethnic basis in Germany. Accordingly, there is also no official data available for Germany in this regard. The reason for this is the persecution of ethnic minorities during the National Socialist tyranny. This is also explained by the Federal Statistical Office of Germany (frequently asked questions: www.destatis.de/DE/Themen/Gesellschaft-Umwelt/Bevoelkerung/Migration-Integration/_inhalt.html).
Population characteristics	We assessed and report self-reported data on age (Mage = 46.62 ± 17.97, range: 18-89), gender (61.7% women), completed years of education (Myears = 16.33 ± 2.26, range: 10-20) , monthly net household income (median income category= 3,000-5,000€) and household wealth (median wealth category = 33,000-142,999€).
Recruitment	The survey was part of the Konstanz Life-Study, a well-established, large-scale study in Konstanz, Germany. Participation in this on-site study was voluntary. Participants were recruited via various approaches including print and online articles in a regional newspaper, household advertising with the delivery of a study flyer to private households in all districts of the city of Konstanz, billboards in the Konstanz area, as well as study advertisements in the university newsletter and Instagram channel. Employees of the town hall and the district administration office were given permission by their employer to take part during working hours. In addition, participants from previous waves of the Konstanz Life-Study were invited by email to take part. In order to make the study easily accessible, appointments for participation could be booked online, by telephone and also in person on-site. This broad range of recruitment strategies aimed at reaching different populations within Konstanz, especially people of all age groups and from different socio-economic backgrounds. As the survey was part of the Konstanz Life-Study, participants received feedback on measured health parameters and we offered the opportunity to take part in a lottery as compensation. Self-selection bias cannot be completely ruled out, as participants voluntarily participated in the study. However, bias with regard to the topic of the presented analyses seems unlikely, as recruitment information provided general information about the Konstanz Life-Study (see also here https://www.uni-konstanz.de/life-studie) and did not emphasize sustainability, climate change or carbon inequality.
Ethics oversight	The study was approved by the ethics committee of the University of Konstanz (ID number: 10/2016). The declaration is provided by the authors upon request.

Note that full information on the approval of the study protocol must also be provided in the manuscript.

Field-specific reporting

Please select the one below that is the best fit for your research. If you are not sure, read the appropriate sections before making your selection.

☐ Life sciences ☒ Behavioural & social sciences ☐ Ecological, evolutionary & environmental sciences

For a reference copy of the document with all sections, see nature.com/documents/nr-reporting-summary-flat.pdf

Behavioural & social sciences study design

All studies must disclose on these points even when the disclosure is negative.

Study description	The present survey was part of the Konstanz Life-Study, an on-site community study assessing the health and fitness as well as self-reports including attitudes and behaviors of citizens from the Konstanz region (see also here https://www.uni-konstanz.de/life-studie). The study location was in a public building in the city center of Konstanz. Quantitative survey data were collected.

Research sample	The present sample includes data from 1,386 participants (Mage = 46.62 ± 17.97, range: 18-89; 61.7% women) from the Konstanz region. Data was collected as part of the Konstanz Life-Study, and participation was voluntary. The sample is not fully representative of the German population. We report relevant demographic information to facilitate a comparison with national statistics. For example, the median wealth in our sample is comparable to the German national median net wealth.
Sampling strategy	As an on-site study, the maximum participant capacity was 1,500. We used a convenience sampling approach. Recruitment focused on the local Konstanz region and employed a wide range of recruitment strategies to reach diverse populations (as described in "Recruitment"). This is reflected, for example, in the wide age range of our sample. The median wealth in our sample is comparable to the German national median net wealth. We based our sample size on comparable studies and aimed for a minimum of 1,000 participants.
Data collection	Between March 6 and April 29, 2023, a total of 1,415 participants completed a questionnaire on-site under the supervision of trained study staff, either using a computer-based questionnaire using the software Unipark (n = 1,220) or a paper-and-pencil version (n = 166). There were no experimental conditions.
Timing	Data was collected between March 6 and April 29, 2023.
Data exclusions	Survey data from 1,415 participants was collected. Out of these, 29 participants were excluded due to missings on core variables for the analyses.
Non-participation	Out of 1,442 participants registered for the study, 27 participants did not fill in the questionnaire including the measures analysed in the present manuscript.
Randomization	The study included neither experimental conditions nor a randomization component.

Reporting for specific materials, systems and methods

We require information from authors about some types of materials, experimental systems and methods used in many studies. Here, indicate whether each material, system or method listed is relevant to your study. If you are not sure if a list item applies to your research, read the appropriate section before selecting a response.

Materials & experimental systems

n/a	Involved in the study
☒	☐ Antibodies
☒	☐ Eukaryotic cell lines
☒	☐ Palaeontology and archaeology
☒	☐ Animals and other organisms
☒	☐ Clinical data
☒	☐ Dual use research of concern
☒	☐ Plants

Methods

n/a	Involved in the study
☒	☐ ChIP-seq
☒	☐ Flow cytometry
☒	☐ MRI-based neuroimaging

Plants

Seed stocks	-
Novel plant genotypes	-
Authentication	-
